# Transcriptome-Based Differentiation of Closely-Related *Miscanthus* Lines

**DOI:** 10.1371/journal.pone.0029850

**Published:** 2012-01-10

**Authors:** Philippe Chouvarine, Amanda M. Cooksey, Fiona M. McCarthy, David A. Ray, Brian S. Baldwin, Shane C. Burgess, Daniel G. Peterson

**Affiliations:** 1 Institute for Genomics, Biocomputing and Biotechnology, Mississippi State University, Mississippi State, Mississippi, United States of America; 2 College of Veterinary Medicine, Mississippi State University, Mississippi State, Mississippi, United States of America; 3 Department of Biochemistry, Molecular Biology, Entomology and Plant Pathology, Mississippi State University, Mississippi State, Mississippi, United States of America; 4 Department of Plant and Soil Sciences, Mississippi State University, Mississippi State, Mississippi, United States of America; United States Department of Agriculture, United States of America

## Abstract

**Background:**

Distinguishing between individuals is critical to those conducting animal/plant breeding, food safety/quality research, diagnostic and clinical testing, and evolutionary biology studies. Classical genetic identification studies are based on marker polymorphisms, but polymorphism-based techniques are time and labor intensive and often cannot distinguish between closely related individuals. Illumina sequencing technologies provide the detailed sequence data required for rapid and efficient differentiation of related species, lines/cultivars, and individuals in a cost-effective manner. Here we describe the use of Illumina high-throughput exome sequencing, coupled with SNP mapping, as a rapid means of distinguishing between related cultivars of the lignocellulosic bioenergy crop giant miscanthus (*Miscanthus × giganteus*). We provide the first exome sequence database for *Miscanthus* species complete with Gene Ontology (GO) functional annotations.

**Results:**

A SNP comparative analysis of rhizome-derived cDNA sequences was successfully utilized to distinguish three *Miscanthus × giganteus* cultivars from each other and from other *Miscanthus* species. Moreover, the resulting phylogenetic tree generated from SNP frequency data parallels the known breeding history of the plants examined. Some of the giant miscanthus plants exhibit considerable sequence divergence.

**Conclusions:**

Here we describe an analysis of *Miscanthus* in which high-throughput exome sequencing was utilized to differentiate between closely related genotypes despite the current lack of a reference genome sequence. We functionally annotated the exome sequences and provide resources to support *Miscanthus* systems biology. In addition, we demonstrate the use of the commercial high-performance cloud computing to do computational GO annotation.

## Introduction

Nucleic acid-based identification techniques are used to improve agronomic species through molecular breeding and/or transgenesis. Moreover, the ability to genetically identify and distinguish between related species, cultivars/strains, and individuals is central to technology commercialization and the protection of intellectual property [1]–[3]. While a number of restriction site polymorphism-, random amplicon-, and repeat polymorphism-based molecular marker techniques have been developed to compare individuals and construct linkage maps [4], Illumina sequencing makes it affordable to conduct robust assays at the much higher resolution of single nucleotide polymorphisms (SNPs) [5], [6]. SNP assays relying on whole genome sequence comparisons are not currently affordable for practical use in commercial settings and for agricultural patents. Moreover, the very large numbers of SNPs in the non-coding regions of genomes, which tend to be under relatively low evolutionary constraint, provide much larger datasets than needed for most mapping and identification/differentiation projects. Exome screening based on high-throughput sequencing, however, is a potential method for comparison of evolutionarily constrained sequences.

Giant miscanthus (*Miscanthus × giganteus*), a fast-growing perennial grass that originated in Japan [7], is a hybrid between the diploid *Miscanthus sinensis* (2*n* = 2*x* = 38) and the tetraploid *M. saccharifloris* (2*n* = 4*x* = 76). Its seed sterility (propagation is traditionally via rhizome cuttings), non-invasive nature, efficient C4 metabolism (particularly at cold temperatures), deciduosity, low nutritional requirements, high photosynthetic output, and ability to grow on marginal lands have made it among the most promising dedicated lignocellulosic bioenergy feedstocks [8], especially in areas such as the U.S. and Europe where it has no close wild relatives [9]. Despite the potential of giant miscanthus as a bioenergy crop, very little is known about the molecular mechanisms underlying its basic biology.

Although, giant miscanthus is closely related to sugarcane and sorghum [10], the lack of dedicated functional genomics resources for these three species is a bottleneck for understanding molecular processes underlying the bioenergy qualities of these crops. This lack of molecular genetic data not only hinders strategies aimed at improving giant miscanthus, but it also makes it difficult for plant breeders to prove whether new varieties that they have discovered or developed are genetically different from existing varieties.

Recently, Swaminathan et al. [11] conducted genome survey sequencing and small RNA sequencing in giant miscanthus. Their research revealed that repetitive sequences dominate the giant miscanthus genome. Moreover, the coding regions of the giant miscanthus genome are similar to coding regions in other grasses. Additionally, most small RNAs appear to be the products of transcribed repeats.

Here we describe the use of high-throughput exome sequencing as a means of distinguishing *Miscanthus* × *giganteus* cultivars and *Miscanthus* species. The approach is applicable to technology commercialization, plant improvement, molecular genetic mapping, and phylogenetics. We constructed a first draft of the *Miscanthus* exome from transcript contigs built from cDNA reads of all seven plants utilized in this study. These transcripts were functionally annotated using the Gene Ontology (GO), and the data is publicly available via AgBase [12] (http://www.agbase.msstate.edu).

## Results and Discussion

### Plant Materials

Seven different plants were utilized in this study. Three of the plants were believed to represent the *Miscanthus × giganteus* cultivar ‘Freedom’. We designated the ‘Freedom’ plant first provided to us as FO for ‘Freedom’, original; the other two ‘Freedom’ plants were obtained from a field and a nursery, and thus designated FF and FN, respectively. Two plants representing the *Miscanthus × giganteus* cultivars ‘Illinois’ (I) and ‘Canada’ (C) were also included in the study as was a plant labeled *Miscanthus floridulus* (F). Based upon its physical appearance and growth, the F plant was suspected of actually being *Miscanthus × giganteus*. Of note, misidentification and mislabeling of *Miscanthus* species is common [7]. In addition a diploid *Miscanthus sinensis* plant (MS) was used as an outgroup.

### Transcriptome Sequencing

A rhizome was obtained from each of the seven plants described above; rhizomes were utilized because our research was conducted during the winter, and leaf tissue was not available from all genotypes. mRNA was extracted from each rhizome, reverse-transcribed to produce cDNA, and the cDNA was sequenced using an Illumina Genome Analyzer. We chose to sequence cDNAs because coding sequences are evolutionarily constrained by the function of the proteins they encode [13]. Thus SNPs in coding sequences are likely informative of functional genetic divergence. We generated 8.9 million Illumina reads from cDNA populations obtained from rhizomes of the seven different *Miscanthus* plants described above.

### Phylogenetic Analysis

**Figure 1:**
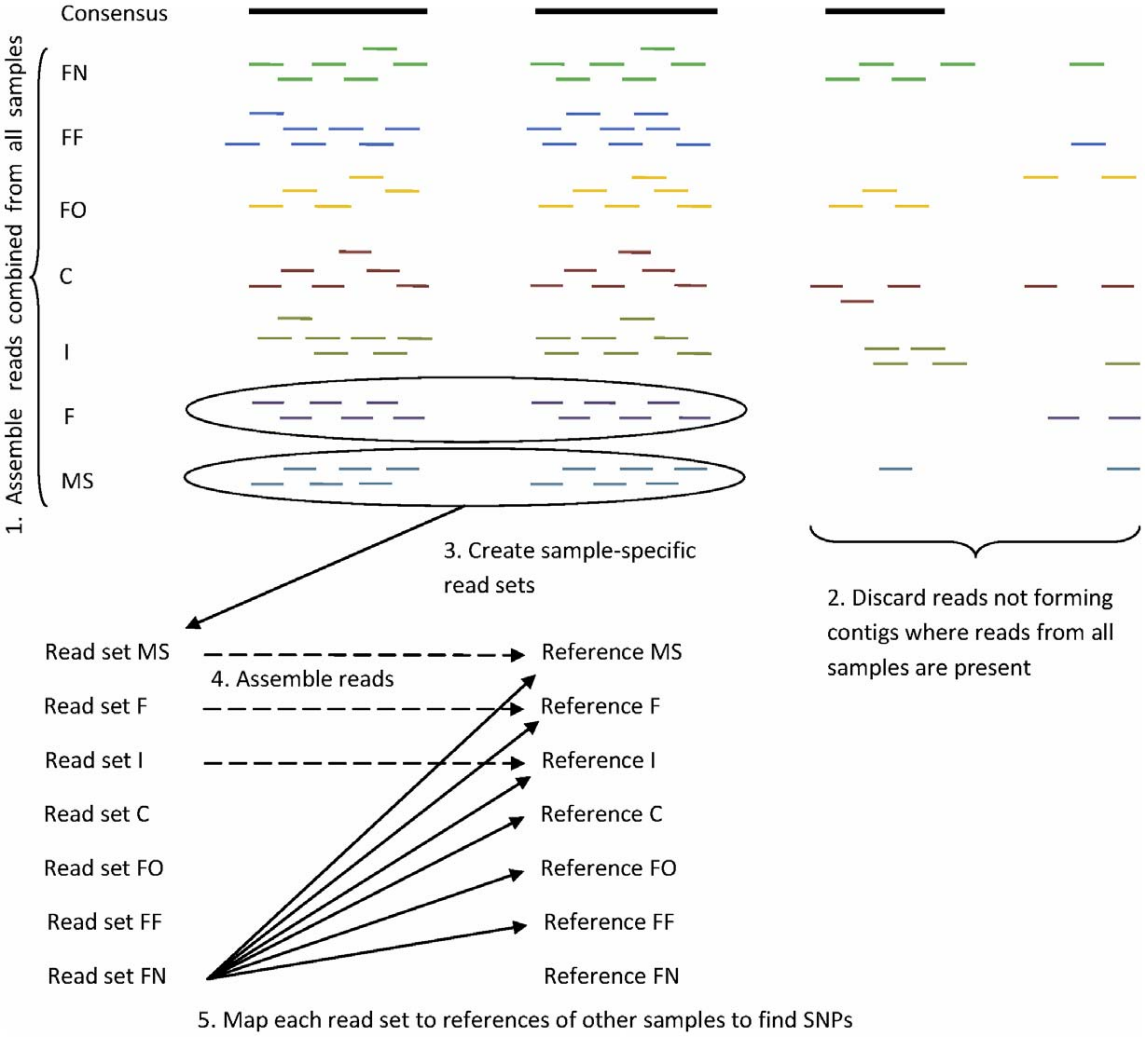
Outline of procedure used to identify SNPs from miscanthus samples.

To describe phylogenetic divergence among all seven samples, we used the method shown in Figure 1. We pooled the sequence reads from all samples and assembled the reads into contigs. For this analysis we needed to identify cDNA regions represented in all samples; therefore, we only considered the reads from the contigs where reads from all seven samples were present (14.64% of all reads).

**Figure 2:**
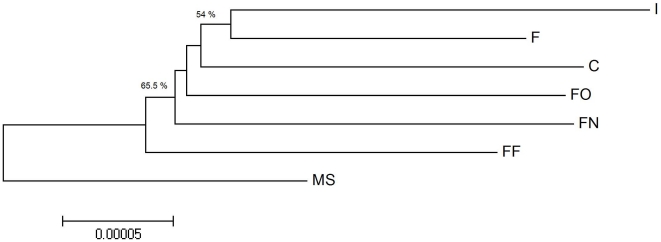
Phylogenetic tree inferred by SNP analysis in common regions of all seven samples. Phylogeny is inferred using weighted SNPs/bp to prepare a distance matrix and generate the neighbor-joining tree for the miscanthus samples.

The reads were then compiled into their sample-specific read sets, which ranged from 33,095 to 370,352 reads. The reads within each read set were assembled into contigs. Common regions in the consensus sequences of these sample-specific contigs were used as references for alignment of reads from each of the other read sets. The sums of lengths of the reference sequences in these read sets ranged from 1,315 to 416,163 bp. The resulting alignments for every pair of samples, e.g., alignment of the FF reads to the FO reference and alignment of the FO reads to the FF reference, allowed us to identify two sets of SNPs for each pair of samples (Table 1). In this case, a SNP is a single nucleotide variation between a reference sequence of one sample and consensus of homologous reads of another sample aligned to this reference sequence. To construct a distance matrix we used weighted SNP/bp values. As mentioned above, the number of reads in different sample-specific read sets varied significantly. Thus, SNPs identified by aligning reads from samples with a low number of reads were underrepresented (a smaller subset of them was identified). Therefore, we utilized counts of SNPs per aligned base, which included bases of every aligned read, rather than SNPs per reference base with alignment. This allowed us to add additional weight to SNPs identified by samples with a low number of reads. For each pair of alignments (e.g., FO vs. FF and FF vs. FO) we calculated the mean number of SNPs/bp (SNPs per aligned base) to construct the distance matrix (Table 2). Each of these mean values represents a normalized measure of genetic variation between the compared samples. A neighbor joining tree inferred from the data is presented in Figure 2. To determine nodal support we performed a bootstrap test as described in the Methods section. The resulting support values, calculated using a Majority Rules approach, are provided in the figure.

**Table 1:** SNPs per aligned bp identified in comparative analysis of cDNA regions common to all samples.

	FF	FO	FN	I	C	F	MS
FF	-	0.000413390	0.000388363	0.000470852	0.000349889	0.000546697	0.000533935
FO	0.000314511	-	0.000348281	0.000434378	0.000309330	0.000486504	0.000502400
FN	0.000319526	0.000370514	-	0.000472350	0.000359891	0.000531350	0.000557107
I	0.000287344	0.000333024	0.000314453	-	0.000306604	0.000462724	0.000500130
C	0.000356861	0.000409450	0.000387226	0.000479916	-	0.000491909	0.000558566
F	0.000102675	0.000137919	0.000125332	0.000182317	0.000112822	-	0.000236819
MS	0.000187104	0.000244045	0.000230092	0.000334766	0.000212052	0.00060301	-

**Table 2:** Distance matrix.

	FF	FO	FN	I	C	F	MMS
FF	-						
FO	0.00036395	-					
FN	0.00035394	0.00035940	-				
I	0.00037910	0.00038370	0.00039340	-			
C	0.00035337	0.00035939	0.00037356	0.00039326	-		
F	0.00032469	0.00031221	0.00032834	0.00032252	0.00030237	-	
MS	0.00036052	0.00037322	0.00039360	0.00041745	0.00038531	0.00041991	-

Our analysis was based on more than 400 million bases of cDNA sequence data from the seven plants. From this data set, we focused on cDNA regions with high quality representation in all seven samples (4.7 million bases total) for SNP analysis. Importantly, the phylogenetic tree constructed from the data exactly represents the known breeding history of the giant miscanthus plants. Of note, a previous AFLP-based approach was unable to demonstrate that sequence differences exist among giant miscanthus cultivars [7] that we differentiated here. Based upon our data, we concluded the following about the seven *Miscanthus* samples:

1. The ‘Freedom’ plants FO, FF, and FN are more similar to each other than they are to ‘Illinois’. On average ‘Illinois’ is 70% less similar to FO, FF and FN than FO, FF and FN are to each other.
2. The mRNA sequence data from FO, FF, and FN are not sequence identical. This could reflect differences in allele/homolog/paralog expression between the ostensibly genetically identical plants. However, the level of variation is very low, compared with the inter-cultivar or interspecies *Miscanthus* comparisons.
3. ‘Canada’ is related to ‘Illinois’ and the three ‘Freedom’ varieties, but it is more similar to the three ‘Freedom’ varieties than it is to ‘Illinois’. ‘Canada’ is most similar to FO followed by FN and then FF.
4. F (the plant labeled *M. floridulus*) is related to all other plants in the analysis, but it groups more closely with the giant miscanthus cultivars (‘Canada’, ‘Freedom’, and ‘Illinois’) than it does with MS. Its similarity to giant miscanthus indicates that F is most likely a mislabeled *Miscanthus* × *giganteus* plant.

Our findings strongly suggest that multiple genotypes of giant miscanthus are available. Genetic differences might account for observed differences in optimal growth region, disease resistance/susceptibility, and yield observed between giant miscanthus cultivars. Planting a single genotype over a large geographic area increases susceptibility of the crop to catastrophic loss [14], [15]. Our study indicates that the three giant miscanthus cultivars studied (*Freedom*, *Illinois*, and *Canada*) are genetically different and that this diversity can be exploited in future cultivar development.

### Exome Assembly

**Figure 3:**
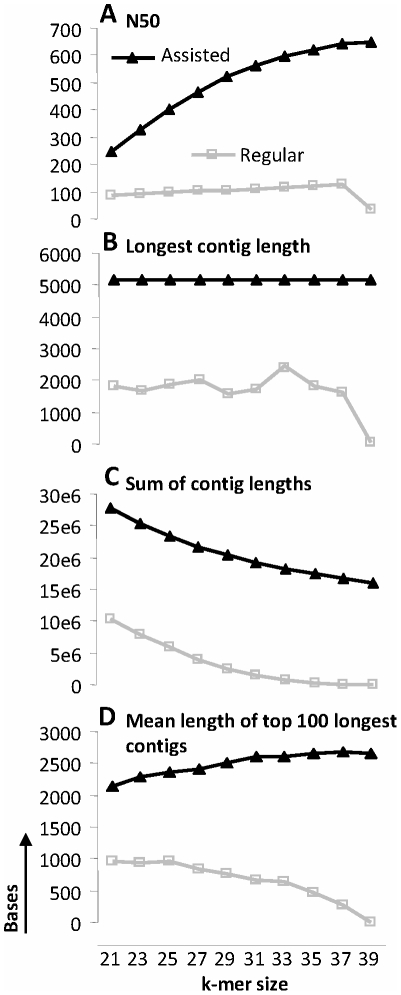
Impact of *k*-mer size on characteristics of *Miscanthus × giganteus* exome assembly in Velvet. Assisted assemblies were assisted with *Sorghum bicolor* transcript references. (A) N50 vs. *k*-mer size. (B) Longest contig length vs. *k*-mer size. (C) Sum of contig lengths, Mb vs. *k*-mer size. (D) Average length of the top 100 longest contigs vs. *k*-mer size.

We also produced two miscanthus exome assemblies by separately assembling *Miscanthus sinensis* reads and combined reads from all varieties of *Miscanthus × giganteus* using Velvet [16]. Velvet contains a module called Columbus that can be used for assisted transcriptome assembly using transcript sequences of a nearby species. *Sorghum bicolor*, a species with a complete genome sequence and extensive transcript sequence resources [17], is closely related to *Miscanthus* [7], and thus we utilized *Sorghum bicolor* in assisted transcriptome assembly of the *M. sinensis* and *M. × giganteus*. Assisted assemblies afforded a significant improvement over non-assisted assemblies as shown in Figure 3. The four graphs represent the effects of varying *k*-mer size on various characteristics of assemblies. For genomic sequence data, the optimal assembly in Velvet is achieved by varying the *k*-mer size to find the maximum N50 and the smallest number of long contigs, while using the expected coverage threshold to minimize misassemblies. This approach is not applicable for transcript assemblies where the number of contigs should ideally be equal to the number of transcripts. For transcript assemblies ideal contig lengths should correspond to actual cDNA lengths and, due to differential gene expression, expected coverage cannot be used. For transcript assemblies, it is more applicable to maximize the contig lengths of longer contigs in the assembly by varying the *k*-mer size. The shorter contig lengths resulting from shorter than optimal *k*-mer length correspond to presence of misassembled transcript fragments. The shorter contig lengths resulting from longer than optimal *k*-mer length correspond to under-assembled contigs due to wasted coverage (unused reads with insufficient overlaps). Velvet outputs only the length of the longest contig (Figure 3, B). However, as shown in this graph, the longest contig in the assisted assemblies of *Miscanthus × giganteus* was not affected by varying *k*. Therefore, we calculated the average length of top 100 longest contigs for every assembly (Figure 3, D). We selected the optimal assemblies by finding a peak in this metric – *k* = 37 for the *Miscanthus × giganteus* assembly and *k* = 23 for the *Miscanthus sinensis* assembly. To validate this method for selection of optimal transcript assemblies, we assembled *Arabidopsis thaliana* transcripts using Illumina RNA-seq reads from NCBI Short Read Archive (ftp://ftp-trace.ncbi.nlm.nih.gov/sra/sra-instant/reads/ByRun/litesra/SRR/SRR018/SRR018346/SRR018346.lite.sra). The reads were assembled using exactly the same assisted assembly pipeline that was applied for the *Miscanthus* transcript assemblies. To estimate quality of each assembly generated by varying the *k*-mer size, we aligned the resulting transcripts to the standard *Arabidopsis thaliana* transcript assemblies downloaded from (ftp://occams.dfci.harvard.edu/pub/bio/tgi/data/Arabidopsis_thaliana/) and calculated the number of bases in the regions where our transcript contig sequences aligned without overlapping each other to the standard transcript sequences with 100% identity. The results are shown in Table 3. As we expected, the maximum of the quality metric described above occurred at the same *k*-mer size (*k* = 19) as the maximum of the average length of the top 100 longest contigs.

**Table 3:** Transcript assembly metrics evaluation using *Arabidopsis thaliana* assemblies.

k	Average length of the top 100 longest contigs	Length of the longest contig	N50	Number of megablast hits with 100% identify to the standard transcript sequences produced by the contig sequences	Number of bases in the regions where our transcript contig sequences aligned without overlapping each other to the standard transcript sequences with 100% identity
15	1261	1957	8	661	8571
17	1482	2365	110	73600	1789362
19	**2028**	4616	223	92409	**2189814**
21	1886	4182	165	73506	2124487
23	1732	5050	235	47372	2040209
25	1662	5048	300	31027	1821088
27	1590	5046	346	20384	1493454
29	1457	5044	379	13093	1102776
31	1382	5042	416	7656	750977
33	1253	4260	474	3679	427093
35	1005	4250	510	1362	120707

The *Miscanthus* transcript contigs identified using Velvet were processed with the *de novo* transcriptome assembler Oases (http://www.ebi.ac.uk/~zerbino/oases/). This analysis identified 29,795 *Miscanthus × giganteus* transcripts and 14,066 *Miscanthus sinensis* transcripts and generated splicing annotation for these transcripts.

### Functional Annotation and Analysis

We did functional annotation of the *Miscanthus* mRNAs using GO. Since these sequences are novel, there is no direct experimental evidence for their function and GO annotation must rely on sequence analysis. The most common type of GO annotation derived from sequence analysis is annotations based on functional motif and domain analysis using InterProScan [18]. Although widely used, InterProScan requires considerable computational power and thus is typically run on clusters. However, a recent trend in bioinformatics is the use of cloud computing for analysis, [19], [20] so we tested the use of the publicly available Amazon EC2 cloud to do functional annotation. This approach provided 58,392 GO annotations for 14,098 miscanthus transcripts, 24,874 transcripts were provisionally GO annotated as “ND”, (i.e., “No Data”), and the remaining 4,881 transcripts could not be annotated using this procedure (e.g. sequence too short to provide reliable results). When transcripts are grouped into gene models, 32% of *Miscanthus* gene models were annotated with non-“ND” GO terms, indicating a predicted function, and 89% of *Miscanthus* gene models were annotated counting GO terms with the “ND” evidence – these will have to await experimental characterization of function. In comparison, 58% of sorghum genes have GO annotation (based on the current GO Consortium release). Since sorghum gene products are mostly annotated using the same method as we used for *Miscanthus*, we can conclude that our transcript assemblies afforded functional annotation of a comparable percentage of gene products to that of another mostly computationally annotated plant species. Using InterProScan on the Amazon EC2 cloud resulted in the average speed of 3 h 9 min per 1,000 nucleotide sequences (with the average sequence length of 570 bp) at a cost of $21.39 per 1,000 nucleotide sequences. However, mappings from InterPro functional domains to GO are revised on a monthly basis and corresponding GO annotations also need to be updated and this will add to the cost of GO annotation.

We are also providing manually derived GO annotation by transferring annotations from closely related sequences (based on sequence alignments) that have experimentally derived GO annotations [12]. This approach identified 57 GO annotations for eight transcripts. Manual biocuration of plant species within the GO Consortium has focused on the model plant *Arabidopsis thaliana* [21] and, more recently on cereals such as rice and maize [22]. Notably, although *Sorghum bicolor* is closely related to miscanthus, there is currently no experimentally derived GO annotation available for sorghum gene products, so this species was not considered during our manual GO annotation process. This example emphasizes the importance of funded efforts to provide experimentally derived functional annotation for a diverse range of key genes from economically important species.

**Figure 4:**
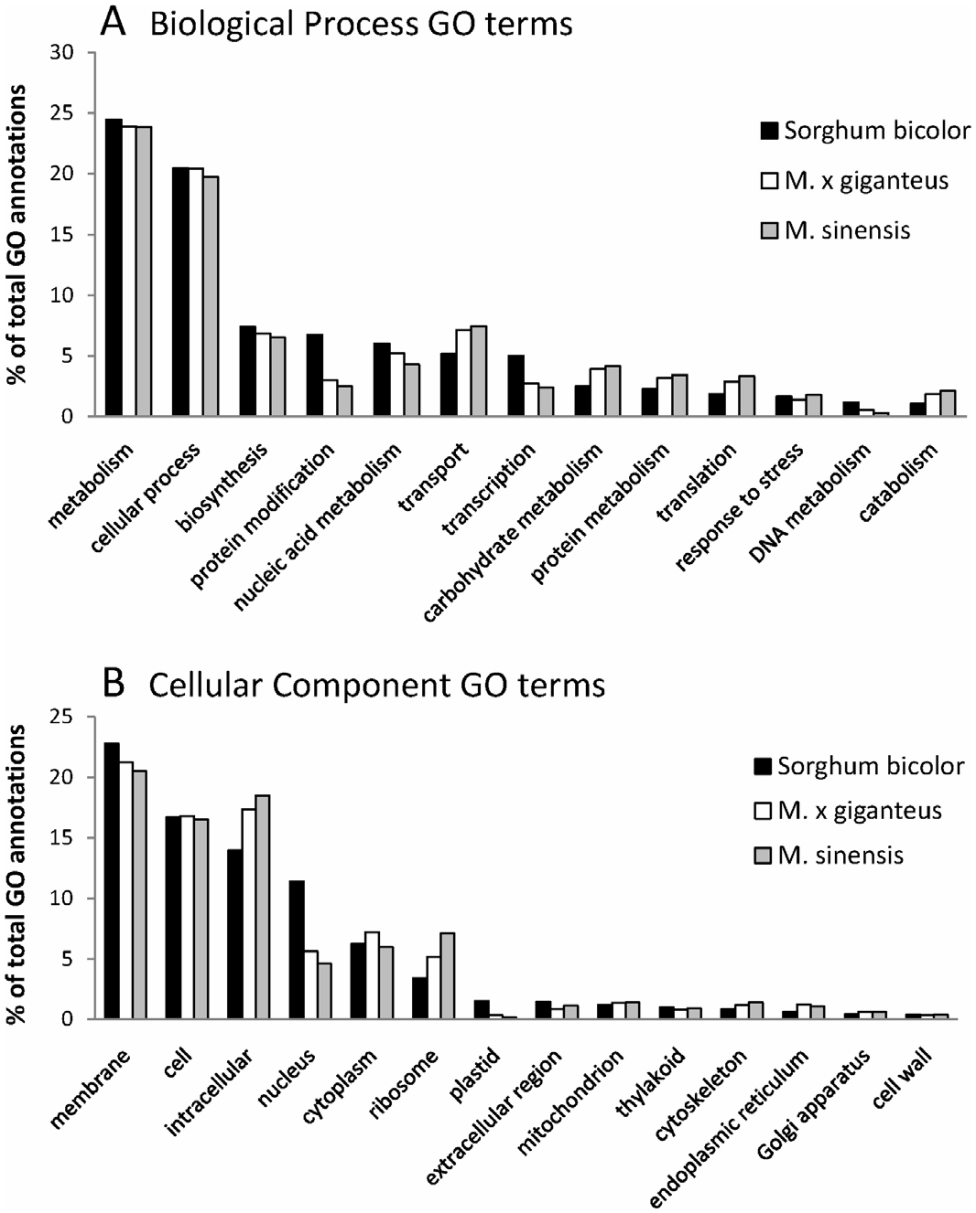
Distribution of GO annotation for miscanthus sequences compared to *Sorghum bicolor*. Sorghum GO annotation was downloaded from AgBase (October 2010) and the Plant GO Slim used to group and compare GO annotations from miscanthus and *Sorghum bicolor*, a closely related species. (A) Biological process GO terms. (B) Cellular component GO terms.

We compared our functional annotations to those from the closely related *Sorghum bicolor*. The proportion of *Miscanthus* gene products with GO annotation is generally similar to that of *Sorghum bicolor* (Figure 4), indicating that our transcripts are representative of a comprehensive miscanthus model transcriptome. Interestingly, the proportion of miscanthus transcripts annotated to nucleus, plastid and ribosome was twice that of sorghum, while the proportion of miscanthus transcripts annotated to protein modification and transcription was half of that found in sorghum. While caution should be used in interpreting functional annotations from two different and incompletely annotated species, our result is not unexpected in the context of rhizome tissue used in this study. Since rhizomes grow underground, it is expected that chloroplasts would be underrepresented. Moreover, while rhizomes can be very active tissues, the samples used were taken from prolonged cold storage, which may have inhibited transcription and translation (protein modification) in general.

Overall, the total number of GO annotations for *M. sinensis* and *M. × giganteus* is proportional to the number of identified transcripts for these two organisms. Similarly, the larger number of sorghum annotations reflects the larger number of known sorghum gene products with GO annotation.

### Data

The transcript assemblies, splice annotations, and functional annotations of *Miscanthus × giganteus* and *Miscanthus sinensis* are located at http://www.agbase.msstate.edu/cgi-bin/information/Miscanthus.pl. The Illumina reads used in this project can be downloaded from NCBI Short Read Archive using the accession SRA025019.

## Methods

### Transcriptome Sequencing

Rhizomes were obtained from plants growing in greenhouses or agricultural fields. Individual dormant rhizomes were collected from each of the seven *Miscanthus* clones. Rhizomes were incubated at room temperature on moist paper on a lab bench for 3 days. Small (100 mg) pieces were taken from each rhizome and ground in liquid nitrogen. These pulverized samples were then re-suspended in 1 ml Trizol reagent (Invitrogen) and transferred to ND Pulse tubes (Pressure Biosciences). Samples were processed in a Barocycler (Pressure Biosciences) for 20 cycles of 20 seconds at 35 kpsi followed by 5 seconds at atmospheric pressure. The resulting lysates were passed through QIAshredder columns (Qiagen) according to the manufacturer's protocol. Lysates were phase-separated using the Trizol protocol (Invitrogen). Following addition of isopropanol, RNA was collected on an RNeasy column (Qiagen). Samples were treated with on-column DNase I and washed as per the RNeasy protocol (Qiagen). Each sample was eluted in 30 µl of RNase-free water. Sample quantity and quality were evaluated spectrophotometrically using a Nanodrop (Thermo) and by capillary electrophoresis using a Bioanalyzer (Agilent).

### Library Construction

Starting with 10 µg total RNA, library construction was done using the Illumina mRNA-seq sample prep kit. Total mRNA was sampled using polyA beads, chemically fragmented and randomly primed for reverse transcription and second-strand synthesis. The resulting cDNA was end-repaired and an adenosine residue was added to produce single-A overhangs. Illumina paired-end sequence adaptors were ligated to the cDNA fragments. Fragments with lengths of approximately 200 bp were sampled from a 2% w/v agarose gel and amplified by PCR (18 cycles) according to the Illumina protocol. A capillary electrophoresis-based Agilent Bioanalyzer was used to quantify and confirm the fragment size distribution of each library. One microliter of each 10 nM mRNA-seq library sample was diluted 10 fold and denatured. For each denatured library, 6 µl of the 1 nM content was diluted in hybridization buffer to 6 pM for clustering (Illumina Standard Cluster Generation Kit v2) according to the manufacturer's protocol. Single read sequencing (40 bp) of the clustered flow cell was done using Illumina's SBS chemistry (Illumina Sequencing Kits v3) and SCS data analysis pipeline v2.4. Flow-cell image analysis and cluster intensity calculations were carried out by Illumina Real Time Analysis (RTA v1.4.15.0) software. Subsequent base-calling was performed using the Illumina GA Pipeline v1.5.1 software.

### Phylogenetic Analysis

To analyze phylogenetic relatedness, we identified SNPs that occur in the regions common to all seven samples. To identify the common regions, Illumina reads from all seven samples were combined and assembled with Velvet. Because SNP identification requires high quality assembly, these Illumina reads were pre-processed prior to assembly. Specifically, we noticed 61% of reads had a single N in the last position; these Ns were removed. Any remaining reads containing Ns were removed. We also set the *-max_gap_count* parameter (the maximum number of gap bases allowed for simplification of two aligned sequences, default: 3) in Velvet to 1, to further improve the assembly quality. Contigs containing at least one read from all seven samples were broken down into sample-specific read sets. Each read set was assembled into a group of sample-specific contigs whose consensus sequences were saved in a reference FASTA file. Each group of sample-specific reads was aligned against each of the other six groups of sample-specific reference sequences using MAQ [23]. All samples except for *Miscanthus sinensis* were from triploid organisms. To account for this we used the *-N 3* option with the *maq assemble* command when aligning reads from such organisms. SNPs were identified using MAQ's *cns2snp* and *SNPfilter* utilities with default parameters. SNP counts were used to calculate the mean of weighted SNPs/bp values for each pair of samples allowing construction of a distance matrix (Table 2). This distance matrix was then analyzed using MEGA 4 [24] to generate the neighbor-joining tree shown in Figure 2. Node support was inferred using a bootstrap test adopted for our method. We created 200 bootstrapped datasets for all 42 alignments that we had, followed by calculation of the mean values of SNPs per aligned base to create 200 distance matrices. These 200 replicates were submitted to the ‘neighbor’ executable of the PHYLIP 3.67 package. The resulting trees were then submitted to ‘consense’ to calculate support values.

### Exome Assembly and Functional Analysis

We used Bowtie [25] to create alignments (SAM files) to *Sorghum bicolor* transcripts. The transcripts were downloaded from the Gene Index Project (ftp://occams.dfci.harvard.edu/pub/bio/tgi/data/Sorghum_bicolor/). The reference sequences, SAM files and unmapped reads were used for cDNA contig assembly in Velvet. We used default parameters without setting coverage cutoff or expected coverage. This was done because expected coverage cannot be assessed for gene expression data. Transcripts were identified by processing the resulting contigs in Oases using default parameters.

The identified transcript sequences were functionally annotated to the GO [26] using standard, GO Consortium compliant biocuration techniques [27]. Since these sequences were not associated with any published functional literature they were GO annotated by manual inspection of BLAST alignments to GO-annotated plant genes using the *GOanna* tool [12] and functional motifs and domains were mapped to the GO using InterProScan. InterproScan IDs were then mapped to GO:IDs and the information formatted as a standard gene association file. We compared these results against GO annotation provided for *Sorghum bicolor* obtained from AgBase (October 2010), as both sorghum and *Miscanthus* have only computationally predicted GO annotations. For each species, GO annotations were summarized into major categories using GOSlimViewer (http://agbase.msstate.edu/cgi-bin/tools/goslimviewer_select.pl) with the Plant GOSlim set. GO annotations were quality checked to meet GO Consortium standards and publicly released via the AgBase database.

### Amazon EC2 Cloud Computing

While sequence alignment using MAQ and sequence assembly using Velvet are routinely done using local servers, the InterProScan analysis is extremely CPU-intensive and consequently the program is typically run on a computer cluster. We chose to create an on-demand cluster using 10 high-CPU instances from the Amazon EC2 cloud (http://aws.amazon.com/ec2). InterProScan was installed on an attachable Elastic Block Storage partition. The cluster was started from an instance with the installed StarCluster software (http://web.mit.edu/stardev/cluster/). StarCluster allows specifying an attachable partition available to all cluster nodes via Network File System. We used this feature to make the Elastic Block Storage partition with InterProScan accessible from all cluster nodes. StarCluster also comes with the pre-installed SGE (Sun Grid Engine) queuing system supported by InterProScan. To avoid problems with InterProScan/SGE hanging when processing large files with thousands of nucleotide sequences, we split files into smaller files with up to 1,000 nucleotide sequences, setting the chunk size parameter in InterProScan to 60 and setting the *finished_jobs* parameter in SGE to 20,000. (Increasing the chunk size and the *finished_jobs* parameter allows processing files with longer sequences or a greater number of sequences, but this can decrease the processing speed). For our dataset, this setup resulted in the average speed of 3 h 9 min per 1,000 nucleotide sequences (with the average sequence length of 570 bp) at the cost of $21.39 per 1,000 nucleotide sequences.
